# A Neuropsychological Rehabilitation Framework to Address Cognitive and Neurobehavioral Impairments After Strokes to the Anterior Communicating Artery

**DOI:** 10.3389/fnhum.2022.808011

**Published:** 2022-06-10

**Authors:** Ramiro Cruces, Indhira Muñoz-García, Santiago J. Palmer-Cancel, Christian Salas

**Affiliations:** ^1^Clinical Neuropsychology Unit, Centre for Human Neuroscience and Neuropsychology, Faculty of Psychology, Diego Portales University, Santiago, Chile; ^2^Hospital Metropolitano, Santiago, Chile; ^3^Brain and Behavioral Associates, PC, Albuquerque, NM, United States

**Keywords:** anterior cerebral artery, stroke, neurobehavioral changes, anterior communicating artery, neuropsychological rehabilitation

## Abstract

Patients with strokes to the Anterior Communicating Artery (ACoA) pose an important challenge to rehabilitation teams due to a particular mix of cognitive and behavioral impairments (anosognosia, anterograde amnesia, prospective memory problems, and executive dysfunction). These deficits often compromise engagement with rehabilitation, learning and generalization. The goal of this article is to describe the long-term presentation of a patient with an ACoA stroke (Mrs. B, a 60-year-old electric engineer) as well as her rehabilitation needs and the many challenges experienced by the rehabilitation team when attempting to facilitate functional, vocational and psychosocial recovery. Based on this case, and the existing literature, a neuropsychological rehabilitation framework to understand and address the specific problems and needs of this population is proposed. This framework demands rehabilitation teams to consider: the slow pattern of recovery of this population, the interaction between cognitive and behavioral impairments, the relevance of physical and social environments, the value of personal projects and the need to include psychological and relational interventions.

## Introduction

**FIGURE 1:**
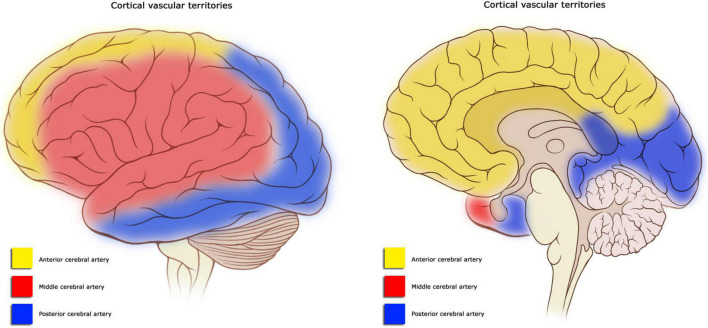
Territory irrigated by the Anterior Cerebral Artery (ACA). The ACA irrigates the medial surface of the brain and the upper border of the frontal and parietal lobes.

**FIGURE 2:**
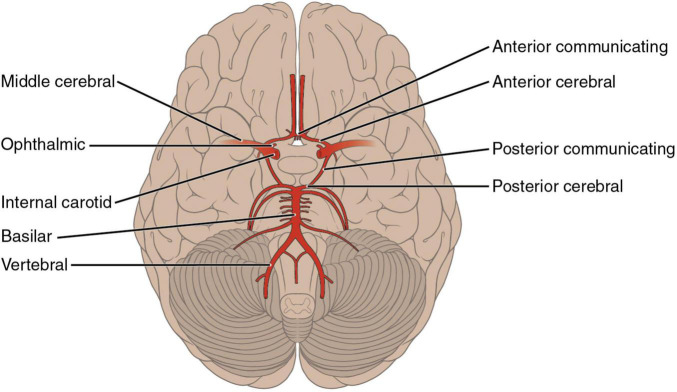
The Anterior Cerebral Artery (ACA) and Anterior Communicating Artery (ACoA).

It is well-known that strokes to different arteries and vascular territories can generate specific neuropsychological and neurobehavioral syndromes. Strokes to the Anterior Cerebral Artery (ACA) are less prevalent than other cerebrovascular events (Nagaratnam et al., 1998). According to Ng et al. (2007), ACA strokes are one of the least common (5%) in comparison to brain infarcts in the medial cerebral artery (MCA–51%), brainstem (11%), or posterior cerebral artery (PCA–7%). The ACA irrigates the medial surface of the brain and the upper border of the frontal and parietal lobes (Berman et al., 1980; see Figure 1). The ACA is often divided into two segments, A1 and A2. The first one extends from the origin of the ACA to its union with the contralateral side by way of the ACoA. The A2 segment is the one distal to the ACoA and runs into the interhemispheric fissure around the genus of the corpus callosum. The ACA gives rise to different branches that irrigate the dorsal aspect of the hypothalamus, optic chiasm, and the medial striate artery, which supplies part of the internal capsule and caudate nuclei and putamen. It also supplies the fornix and the septum pellucidum with help from the pericallosal arteries. The hemispheric branches supply frontopolar, orbitofrontal, internal frontal, paracentral, and internal parietal branches of the medial surface of the hemisphere (Love and Biller, 2007; see Figures 1, 2). Despite the low prevalence of ACA strokes, they can cause important disability due to motor, cognitive and behavioral impairments (Nagaratnam et al., 1998; Kumral et al., 2002; Kobayashi et al., 2011).

Nagaratnam et al. (1998) reported that patients with ACA strokes typically present motor problems, mutism, delayed initiation, reduced fluency, apathy, and overall frontal-lobe dysfunction. Kumral et al. (2002) found acute confused states, mutism, abulia, impaired verbal fluency, hemiparesis, sphincter incontinence, and abnormal reflexes. Bilateral strokes of the ACA can cause primitive reflexes, parkinsonian gait, tremor, and facial dystonia (Kobayashi et al., 2011). Frontal lobe behavioral impairments–such as disinhibition- are more common in survivors with ACA bilateral lesions. Lesions to a specific area of the ACA, the Anterior Communicating Artery (ACoA), can generate a particular set of neuropsychological and neurobehavioral deficits: severe anterograde amnesia, confabulation, dysexecutive problems, anosognosia, and personality change (Logue et al., 1968; Deluca, 1992). More recent studies have supported early observations, describing significant cognitive impairments following ACoA lesions (Nassiri et al., 2018; Ma et al., 2021). Beeckmans et al. (2020) reported that patients who underwent surgical clipping of the ACoA had more deficits in attention, memory, and executive functions compared to a control group.

Even though many patients with damage to the ACoA survive, and positively evolve from a neurological and motor point of view, the residual cognitive and behavioral deficits, as well as emotional and personality changes, have a profound impact on their capacity to function independently and return to premorbid activities. A limitation of the existing literature is that most studies tend to focus on the cognitive and behavioral impairments caused by ACoA strokes during the acute phase. There is only one article, with a very small sample (*N* = 10), that reported a mild degree of cognitive impairment in the chronic phase and a negative association between cognitive dysfunction and community integration (Ravnik et al., 2006). This contrasts with the growing clinical (Winson et al., 2016), and evidence-based knowledge (Evans, 2003; Wood and McMillan, 2017), regarding the cognitive and behavioral rehabilitation of people with diverse forms of executive impairment, memory deficits, and neurobehavioral problems after frontal lobe damage.

The goal of this article is to address this gap by (a) describing the long-term presentation of a patient with an ACoA stroke, as well as her rehabilitation needs, and the many challenges experienced by the rehabilitation team when attempting to facilitate functional, vocational, and psychosocial recovery; (b) to propose a neuropsychological rehabilitation framework to understand and address the specific problems and needs of this population.

## Case Description

Mrs. B is a 65-year-old woman. At the moment of her stroke, she worked as a chief engineer at a wind energy project. She was married and had five children. She lived with her husband and two sons. In 2017 Mrs. B was hospitalized due to a hemorrhagic stroke caused by an aneurysm of the left ACoA. Days after discharge she required a new intervention to clip an aneurysm of the left ACoA. She was sent home 3 days after this second hospitalization. Mrs. B had a quick motor recovery after these events. According to the discharge report from the hospital, she had memory difficulties, confabulations, and temporal disorientation. No language, sensory, or motor problems were observed.

Four months after discharge, she was referred for a neuropsychological evaluation. Her profile of difficulties and strengths showed a dissociation between psychometric and observational/ecologic results. Her performance in all psychometric tasks was within normal range, except for verbal memory (Word Version of the Free and Cued Selective Reminding Test) and multitasking (Behavioral Assessment of the Dysexecutive Syndrome, the Modified Six Elements Test), where she presented with a marked impairment. When psychometric and observational data were integrated, a profile characterized by a marked anterograde memory impairment was observed. At the moment of the assessment, no confabulations were present. Prospective memory was also compromised, so Mrs. B struggled to remember future events and commitments. In relation to executive functions, using Stuss’ Model (Stuss, 2011), Mrs. B presented impairments in energization–capacity to internally initiate, and sustain, a voluntary or non-reflexive response–and monitoring–acknowledging the changes generated by the injury. She was unaware of her difficulties and responded with perplexity and incredulity when confronted by her family and rehabilitation professionals. Notably, other cognitive abilities were preserved, such as, attention span, selective attention, processing speed, expressive and comprehensive language, communicative skills, and viso-constructive skills. Regarding executive abilities, working memory, mental flexibility and abstraction were preserved as well (see Table 1). Due to these difficulties Mrs. B exhibited a drastic decrease in her ability to independently carry out activities of daily living. According to the Activities of Daily Living Questionnaire and Technology (ADLQ-T) (Muñoz-Neira et al., 2012), Mrs. B exhibited a 41% decrease in her level of independence–a 29% decrease is the cut off score for a significant change in the Chilean population. The most compromised areas were work and leisure (33% of change), managing money and shopping (66%), transport in familiar and non-familiar environments (75%) and technology use (86%). According to her family, Mrs. M was not able to sustain a daily routine and take charge over the management of the house, particularly due to a decrease in motivation, flexibility and memory failures. She could become repetitive when attempting to do common tasks at home (e.g., washing the dishes twice) as well as tasks from her previous job (e.g., reviewing maps from a project). She also became disorganized and repetitive when confronted with old and new problems (e.g., selecting what to wear, reorganizing files in an office to create her own workstation). At home, she struggled remembering what to buy and often purchased unnecessary items from the supermarket. When traveling in the community she could easily get lost and disorientated, requiring supervision. Regarding the use of technology, she was unable to use her email, could not remember passwords, use ATMs or computers. Due to these functional difficulties, she was unable to resume her previous job. Nevertheless, Mrs. B was independent in basic activities of daily living such as eating, dressing up, personal hygiene and appearance, and medication intake.

**TABLE 1:** Mrs. B neuropsychological profile.

Cognitive function	Instrument	Raw score	Z score
Global cognitive efficiency	ACE III total score	91/100	0.01
	ACE III attention	18/18	0.7
	ACE III memory	19/26	–0.87
	ACE III verbal fluency	12/14	0.19
	ACE III language	26/26	0.69
	ACE III visuospatial	16/16	0.88
Attention and speed processing	WAIS IV–Direct Digit Span	7	1.35
	Trail Making Test A	33	0.92
	D2 total responses	447	0.52
	D2 concentration	185	1.03
	D2 number omissions	3	1.28
	D2 number commissions	0	0.84
Verbal memory	Grober and Buschke Test-Verbal (total recall)	28	–5.6**
	WMS III–immediate auditive memory index	91	–0.58
	WMS III–delayed auditive memory index	93	–0.49
Visual memory	Rey-Osterrieth Complex Figure Test (delayed recall)	14	–0.15
Visual construction	Rey-Osterrieth Complex Figure Test (copy)	36	0.99
Executive functions	Trail Making Test B	47	1.38
	Verbal fluency letter F	15	0.67
	Verbal fluency letter A	16	1.13
	Verbal fluency letter S	17	1.63
	Verbal fluency animals	16	–0.32
	Hayling test A time	0.9	0.89
	Hayling test B time	4.42	0.44
	Hayling test B score	1.06	–0.56
	WCST N° of errors	16	0.61
	WCST N° of perseverative responses	10	0.41
	WCST N° of perseverative errors	5	0.91
	WCST N° of non-perseverative errors	11	–0.07
	DKEFS design fluency–condition 1	6	–0.66
	DKEFS design fluency–condition 2	7	–0.66
	DKEFS design fluency–condition 3	7	0.33
	DKEFS design fluency–total	27	–0.33
	DKEFS design fluency–filled and empty dots	16	–0.66
	WAIS IV–inverse digit	10	1.66
	WAIS IV–working memory index	124	1.64
	Digit Span Sequencing	10	1.66
	BADS Modified Six Elements Test	Profile 2	–1.9**
	WAIS IV–analogies	28	1.33
Social cognition	The Eyes Test Baron-Cohen	33/36	1.97

ACE-III, Addenbroke’s Cognitive Examination, Chilean Version; WAIS IV, Wechsler Adult Intelligence Scale IV; WMS III, Wechsler Memory Scale III; WCST, Wisconsin Card Sorting test; DKEFS, Delis-Kaplan Executive Function System; BADS, Behavioral Assessment of Dysexecutive Syndrome. **impaired.

In relation to the psychological impact of the stroke, it is interesting to note that Mrs. B did not report any signs of a reactive mood disorder. According to Patient Health Questionnaire-9 (Baader et al., 2012), Mrs. B only reported one depressive symptom (apathy) not fulfilling the criteria for a depressive disorder. She also completed the Beck Anxiety inventory (Beck and Steer, 1993) reporting low levels of anxiety, and the General Scale of the Quality of Life After Brain Injury Questionnaire (Steinbüchel et al., 2010) endorsing a decrease in satisfaction with her current situation and future prospects. During the clinical interview, she presented herself with anosognosia and anosodiaphoria. These observations were systematically explored using different tools, such as the comparison of deficits reported by the patient and her family (Sherer and Fleming, 2014), the Awareness Questionnaire (AQ) (Sherer et al., 1998), and the Dysexecutive Questionnaire (Wilson et al., 1996). She had a vague notion regarding what happened to her, only recognizing that “something broke inside the head.” When asked to elaborate further, she often commented: “they tell me I had a stroke, but I don’t notice anything different in me. I feel ok. I feel capable. This has been a time to relax, but now I need to do something more than staying at home.” When rehabilitation professionals and relatives confronted her with memory and other cognitive issues, she tended to minimize the relevance of such difficulties: “My memory has never been great. Besides, what I do at home hardly demands any effort, so it is difficult to realize I am struggling remembering.”

Despite her unawareness, at times, Mrs. B was able to grasp that after the stroke something had changed in her and she was no longer the same person. She described her pre-injury self as an active and hyper-organized engineer that coexisted with a housewife who orchestrated everything around her. After the stroke she portrayed herself as “more passive,” something that her sons and daughter also reported–but interpreted as laziness and lack of motivation to improve. The family consulted several psychiatrists concerned by this change, believing that Mrs. B was depressed. However, this was not the case since she had no depressive mood or depressive cognitions. This mixture of personality changes and lack of awareness generated important conflicts between Mrs. B and her family. She was often criticized by some of her children because of her lack of initiative and not following a set daily routine. She resented such criticism: “I used to give orders at home before and now everybody tells me what to do. My nature is that no one gives me instructions and now everyone tells me what to eat or what to do. It is like they believe I am stupid now, and I feel I disappoint them since I am no longer the one I was.”

## Rehabilitation

### First Phase of Rehabilitation

Based on findings from the assessment several goals were negotiated with Mrs. B and her family for the first phase of treatment: (a) Psychoeducation to the patient and relatives regarding cognitive and behavioral changes; (b) Increase the frequency and consistency use of memory aids; (c) Increase adherence to a routine that included home-based and community-based activities. A clinical neuropsychologist (RC) and an occupational therapist visited Mrs. B once a week for over 9 months.

*Psychoeducation* has been defined as a process where information about the consequences, prognosis, and management of a medical or psychiatric condition can be delivered (Ekhtiari et al., 2017). Psychoeducation is an evidence-based intervention (Cheng et al., 2014; Ostwald et al., 2014; Kontou et al., 2021; Venkatesan and Ramanathan-Elion, 2021) commonly used in the rehabilitation of people with diverse forms of brain damage (Winson et al., 2016). However, psychoeducation can be a challenging endeavor in patients like Mrs. B, who exhibit a particular set of cognitive impairments (anosognosia, memory difficulties and executive problems) that can compromise the acquisition and acceptance of information. Mrs. B used to say: “I realize and understand what you say, but I just can’t feel it.” When properly scaffolded during conversation she could become temporally aware of some behavioral changes (“I feel off”) but such knowledge was transient. Following the guidelines proposed by Winson et al. (2016), information regarding the neuropsychological profile of ACA strokes, and its impact on memory, behavior, and everyday life, was systematically presented to Mrs. B and her family. Based on the work of Roberts et al. (2006) the observation of Mrs. B’s neuroimages was used as a tool to increase awareness of the stroke and its consequences. In order to promote the consolidation of this information, a therapy notebook was employed to register relevant information discussed during psychology sessions. In addition, the structure of sessions was arranged to promote learning and awareness; each session began revising what had been discussed previously and reminding her about the goal of the session and treatment in general. Relatives were educated in relation to specific strategies to manage memory and energization problems (Winson et al., 2016)- structured routines, alarms, memory systems-, thus minimizing its impact in everyday life (Winson et al., 2016). It is important to note here that psychoeducation was an intervention that extended throughout the whole treatment, particularly due to Mrs. B anosognosia, the psychological difficulties of relatives in understanding and accepting cognitive and behavioral changes in Mrs. B and because of the relational impact the injury had in family bonds.

In relation to the second goal (*memory aids*) following the guidelines presented by Wilson (1996) and Fish and Brentnall (2017), Mrs. B was trained in the use of a memory system that included a mobile phone, a daily planner, and a wall calendar to remember daily and future events. A notebook was also employed to register daily events and facilitate episodic and prospective memory (see Supplementary Appendix 1). Relatives were trained in how to externally support the use of these aids in everyday activities since Mrs. B struggled internalizing them. Mrs. B was very reluctant to use alarms, despite the fact she regularly forgot activities and commitments. Due to prospective memory issues, she often forgot setting alarms, and because of energization problems, she did not initiate such behaviors. Interestingly, certain appointments–related to work and health- mobilized her enough to independently use compensatory tools. This contrasted with activities related to running the household, an activity she considered non-relevant and disconnected to her pre-injury identity. Such observations offered valuable information for the second phase of the treatment.

As for the third goal (*adherence to a routine*), despite the efforts of the rehabilitation team and relatives, Mrs. B did not manage to automatize a daily routine at home and integrate her compensatory tools to organize it. She used to sleep till late in the morning and could become stuck on a task for a long period of time. She tended to engage in household chores that were appealing to her, leaving unattended those she considered as non-interesting, but were necessary. She did not check her diary or use it to plan the day ahead either, requiring prompts from others to trigger the behavior.

After several months of work, and due to the many difficulties described above, the rehabilitation team and the family decided to change the focus of treatment. Mrs. B was increasingly unhappy with her current life and routine, often complaining about how she did not have anything meaningful to do. Improvements in her capacity to be independent and functional in everyday life were slow and marginal, requiring important levels of external support. Her husband, a mechanical engineer, began taking her to his office, hoping she could help him with some administrative work. Surprisingly, this change in scenario mobilized Mrs. B significantly. The days when she joined her husband at work, Mrs. B got out of bed on time and was quicker in getting dressed and ready to go. She was highly motivated to help and could spend long periods of time engaged in tasks of different sorts (e.g., revising maps and reports, sorting out documents, checking out redaction). The rehabilitation team considered that this change in environment was increasing her initiative, probably because the tasks were similar to the ones she used to do before the injury–more automated. From an identity point of view, this new context appeared to re-connect her with her pre-injury self, Mrs. B the engineer, boosting her motivation and thus compensating for energization problems. In addition, when executive problems emerged at the office (see below), her husband intuitively began to offer small levels of support that allowed her to continue. Based on these promising observations, the team and family decided to direct rehabilitation efforts to the work environment.

### Second Phase of Rehabilitation

The second phase of rehabilitation spans from April 2019 to October 2019. The clinical neuropsychologist visited Mrs. B at her husband’s office once a week. As a first step, it was discussed with Mrs. B and her husband the tasks she was required to do. Initially Mrs. B worked gathering and organizing digital information and files, from hard drives and laptops, of the wind energy project she oversaw before the injury. This had been something she tried to do on her own after the injury, but was unable, spending many hours with little progress. Later she focused on reviewing engineering plans from her husband’s office and checking whether the information displayed on them was similar to the project reports. This task required also revising information regarding quantity of materials for each project and calculating its cost.

The clinical neuropsychologist joined Mrs. B while performing the assigned tasks, in order to observe and analyze her performance, thus detecting potential problems that may need support. Regarding the first task (organizing digital information), Mrs. B’s initial approach was highly dysexecutive; she randomly revised documents and filled an excel file with information. She also tended to become stuck in loops where she returned over and over to the same files, feeling anxious and unsure whether she had correctly covered all the material. Another issue that was observed related to Mrs. B difficulty to continue a task between days. Because of memory impairments, Mrs. B tended to forget what she was doing the day before so she did not know where to start from, repeating steps she had already done. The capacity to sustain a plan that extended in a long period of time, a skill required when reviewing large engineering projects, was greatly compromised. Several strategies were developed to address these difficulties. In relation to her dysexecutive approach to the task, a method to facilitate the revision process was jointly created, thus helping her stay on track and feel confident she had not missed anything. The first step of this method was organizing all the material that needed to be revised (3 hard drives and 1 laptop). Since the amount of information was substantial, subtasks were generated with physical reminders (post it notes) and deadlines (1 week for each source of information). A reviewing strategy was also created to revise each of these sources of information avoiding perseverative loops and developing a continuity between days. The folders contained in each device were printed and a work notebook was introduced. This strategy allowed her to cross mark when a folder had been revised already and summarize in the notebook the work accomplished during the day as well as the tasks that needed to be done the next day. Such a strategy facilitated the continuity of her actions from one day to another. Mrs. B managed to accomplish this task in a period of 2 months and found herself very proud to finally have done it. Such success also proved to her husband that Mrs. B was able to carry out relatively complex tasks if a routine was established and adequate supports were introduced for her memory and executive problems. As a rehabilitation team, we realized that Mrs. B was able to learn a strategy, initially with higher levels of support and later more independently. In other words, we observed a promising automatization and internalization of strategies, with the right tasks and the right context.

**FIGURE 3:**
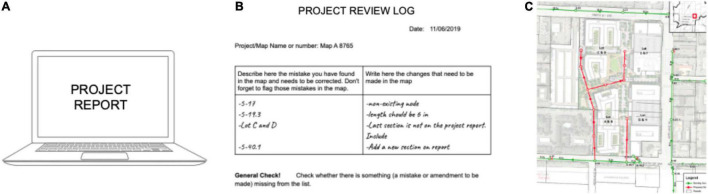
Work set up. Mrs. B used a project log **(B)** to fix and organize information related to the comparison between a map **(C)** and its project report **(A)**. Discrepancies were written down in the log **(B)** as well as necessary amendments. Information about these discrepancies was later passed into a revised version of the project report.

A second project she embarked on was helping her husband with administrative and technical tasks. Previously her husband had asked her to check that engineering plans matched technical and financial reports. This task required to compare both documents and perform calculations regarding quantity of materials and costs. Mrs. B had been unsuccessful initially helping with this task since she often entered into perseverative loops. She was able to correctly analyze an engineering plan and detect errors that needed amendment. However, she did not know how to fix them, or what to do with them, so she had to ask her husband in order to decide what to do next. Importantly, when she later returned to the plan, she found the same mistake without awareness that she had already sorted that element before. A formulation of this problem was built with Mrs. B and it was found that she tended to forget information when comparing plans and reports–a mixture of working memory and divided attention problems. The use of a project log (Supplementary Appendix 2) was introduced in order to register mistakes or inconsistencies that had been detected between maps and reports, thus avoiding perseverative loops (see Figure 3). The use of self-talk was employed as a tool to sustain and manipulate information in mind when comparing maps and reports. For example, Mrs. B learnt to repeat out loud erroneous information that needed to be held in mind and registered in the report: “error in section B, the value must be 3.72”. Because some of these strategies had been employed before, the generalization process was simple and only took a month. Mrs. B managed to maintain her performance in these tasks for months with less support and increased automatization in the use of compensatory strategies.

Four months after initiating her work at her husband’s office, Mrs. B showed significant improvement. She had a richer routine with 3 days attending work and 2 days at home. During workdays her motivation and energy levels were adequate, managing to work for a full day with no visible sign of fatigue. Since she was active, her relatives allowed her to have days off during the week, where her level of motivation and proactivity was not questioned or criticized. According to her husband, although slower than before, Mrs. B was able to effectively help with several administrative and technical tasks. It is remarkable that, despite marked memory and executive problems, Mrs. B was not only able but proactive in using compensatory strategies such as a notebook, calendars, and alarms. This internalization of strategies began to spontaneously generalize to other work and home related tasks, even increasing her awareness of deficits. Thus, she was able to anticipate situations where she foresees that memory or executive problems may occur, implementing compensatory strategies on her own. For example, memory aids became generalized to her living space, something much harder in the first phase in rehabilitation. She began using an organizer at home, would initiate tasks more quickly, would plan ahead for a task, and was using her memory system better. The marked improvement exhibited by Mrs. B in the work setting also helped the rehabilitation team and family to understand the context-dependent nature of her impairments. Her initiation difficulties were less marked when engaged in tasks that were motivating and connected to her pre-injury identity. By closely working with Mrs. B, her husband managed to gain a better understanding of her memory and executive difficulties, as well as the type of tasks and situations that tended to trigger or exacerbate them. He learnt that with some minimal support Mrs. B could become organized and efficient.

Due to an increased sense of efficacy, Mrs. B began to comment that she would like to expand her work into administrative tasks (monitoring income/egress sales). This spontaneous motivation was fueled by her renewed sense of competence and possibly her experience of reconnecting with aspects of her pre-injury sense of self (Mrs. B the engineer and project manager). Unfortunately, due to political events that occurred in Chile during October 2019 the country was paralyzed for months. Immediately after the pandemic arrived and Mrs. B was forced to stay at home. Due to transportation and safety issues the clinical neuropsychologist could no longer visit her. This resulted in an abrupt interruption of the treatment.

## A Neuropsychological Rehabilitation Framework to Work With Anterior Communicating Artery Stroke Patients

Anterior Communicating Artery (ACoA) patients present important challenges to rehabilitation teams and relatives. These challenges tend to relate to marked executive and memory impairments, as well as energization difficulties and anosognosia. Consequently, the first step of rehabilitation, engagement (Ben-Yishay et al., 1985), is often compromised. Episodic and prospective memory deficits contribute to anosognosia and pose important limitations to learn new information and use compensatory methods (Fish et al., 2010; Raskin et al., 2018). Executive problems, such as disorganization and lack of initiation, can also compromise the generation of, and adherence to, a structured routine (Jackson et al., 2014). Furthermore, cognitive and behavioral problems after ACoA strokes are poorly understood by families, often generating negative interactions, and compromising the availability of much needed support. The preservation of motor skills, expressive and comprehensive language abilities, contribute to the difficulty in understanding the nature of these *invisible* deficits (Bracho-Ponce et al., 2021).

**FIGURE 4:**
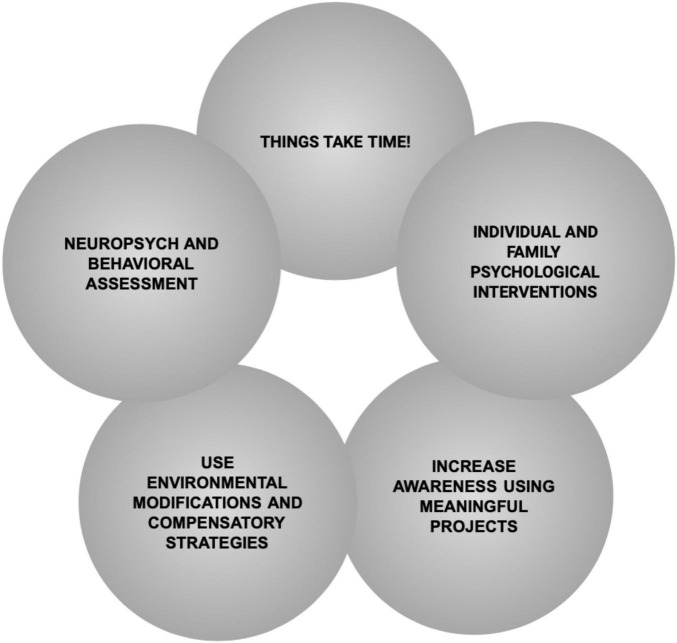
Neuropsychological rehabilitation principles necessary to consider when working with ACoA stroke survivors.

The particular mix of compromised and preserved functions in patients that suffer a stroke of the ACoA demands rehabilitation teams to build complex case formulations that encompass cognitive, emotional, behavioral and identity elements. Due to the interacting nature of these factors, the rehabilitation of this group of patients requires a theoretical framework that guides professionals in addressing the impairments and needs of these patients and their relatives. In this section we would like to propose five principles (see Figure 4) that can help rehabilitation teams to navigate more smoothly the many challenges posed by patients with ACA strokes. These principles have been distilled from our experience working with brain injury survivors like Mrs. B.

### TTT: Things Take Time!

Even though this is an idea that must be put into practice when working with any person that suffered a brain injury (Klonoff, 2010), it is particularly relevant for people with ACoA strokes. As portrayed in the case of Mrs. B, improvements in this population may take months and even years. This has been noted before by the few existing case studies focusing on the rehabilitation of this population (Deluca and Locker, 1996; Stablum et al., 2000; Bolognani et al., 2007). Anosognosia and memory impairments tend to compromise the process by which survivors learn about what happened to them and how impairments affect them in everyday life. As noted by Mrs. B, she understood all the information presented to her by the rehabilitation team, but she felt “normal.” Memory problems also compromised the continuity between sessions, as well as the sedimentation of new information regarding the injury. The use of interventions that tap into procedural learning and preserved skills appears to be a key aspect of the rehabilitation of this population, particularly in terms of introducing a routine with anchors that mobilize behavior or learning to use compensatory strategies (Evans, 2017). However, procedural learning demands time and repetition. A shared understanding (Winson et al., 2016) between the rehabilitation team and relatives is particularly important in this sense, in order to moderate expectations and sustain hope. Besides, a shared understanding of cognitive and behavioral difficulties, and its management, facilitate learning, by allowing the same information to be delivered to the patient by multiple sources (Winson et al., 2016). However, as exemplified in the case of Mrs. B, many deficits presented by patients with ACoA are difficult to understand and accept by relatives. As a consequence, building a shared understanding is a particularly challenging task in this population, requiring time, constant education, and emotional support for relatives. Furthermore, TTT can make sense to rehabilitation professionals, but such approach is not always supported by health care providers that offer specialized support only during early phases of treatment.

### Assessment Must Focus on Both Neuropsychological and Behavioral Impairments

As clearly portrayed by the case of Mrs. B, working with ACoA patients requires building complex case formulations, demanding from rehabilitation teams to have the theoretical and technical competencies to assess and understand the relationship between cognitive, emotional and behavioral impairments (Wilson and Betteridge, 2019). This is particularly relevant since behavioral changes are often overlooked by rehabilitation professionals, or misunderstood as psychiatric problems, for example confounding energization or initiation deficits with depression (Williams et al., 2020). Mrs. B was for a long time misdiagnosed as depressed, missing the link between changes in her behavior and executive problems. In addition, behavioral difficulties are not easy to grasp with traditional assessment tools, since patients with neurobehavioral problems can perform normally on standardized tests but fail in real life situations (Wood and Bigler, 2017). Third party reports can be a useful tool to explore neurobehavioral problems as well as the ecological observations of patients in everyday life. Similar to patients with other types of brain damage, gathering information regarding preserved skills and functions is extremely relevant. These preserved skills are critical when designing interventions. The complex presentation of ACoA patients, and their multiple impairments, demand from rehabilitation teams and families to wisely “pick their battles,” focusing on achievable goals that are relevant for the patient and family, thus promoting motivation, adherence to treatment and self-awareness. The case of Mrs. B is a great example on this matter, showing how the “right” rehabilitation context (work), one connected to the patient’s personal motivations and identity (Mrs. B the engineer), mobilized change and modulated impairments (energization). These observations are consistent with relational paradigms in neuropsychological rehabilitation, which propose that deficits are modulated by physical and social context (Bowen et al., 2010).

### Facilitating Learning and Behavior Activation Through Environmental Modification and Compensatory Tools

Physical and social environments are particularly important for patients with ACoA strokes. Because of episodic and prospective memory impairments, as well as initiation problems, they heavily rely on external aids and cues to trigger desired behaviors. As described in the case of Mrs. B, a notebook was essential in order to scaffold her capacity to remember information from the recent past when performing a complex task. Graphic advance organizers and a Goal-Plan-Review-Routine (Ylvisaker et al., 2005) were also employed to offer cues as a support for organizationally demanding tasks, allowing her to follow a series of steps. The timely and adequate support from her husband was also extremely useful to help Mrs. B overcome situations when she became disorganized or repetitive (“the perseverative loop”). This type of social/external regulation of behavior has been described by Ylvisaker and Feeney (1998) as a collaborative approach in rehabilitation, where interventions for executive impairments are conceptualized as a collaborative enterprise. Structuring a routine has been described as a key intervention in people with marked executive impairments, particularly initiation problems (Evans, 2017). As noted by Jackson et al. (2014), structuring the environment may allow patients to function at a higher level and with less support. However, there is little theorization amongst rehabilitation professionals regarding what structuring the environment means and how to accomplish it. Jackson proposes that structuring the environment requires: (i) routines and rituals bound by time and environmental cues, that orient people into what is next (*anchors*); (ii) physical or social aids that maintain and reassert structure in everyday life or specific tasks (*scaffolding*); (iii) the use of strategies, or well-rehearsed rules, that can guide behavior in simple and complex tasks (*strategies*). According to the same author a structured environment is unambiguous, consistent, predictable, planned and organized, exposes patients to tasks within their capacity, is goal-focused, promotes generalization and offers activities that are personally meaningful. This last point is particularly relevant when using behavioral interventions from a holistic point of view (Feeney and Capo, 2010; Feeney and Gould, 2021), and is clearly exemplified by the case of Mrs. B. Her struggle adhering to a house-bound routine can be explained not only by her particular set of cognitive and behavioral difficulties, but also by the disconnection between those activities and her pre-injury identity.

### Promoting Awareness Through Personal Projects

The rehabilitation of awareness impairments is perhaps one of the most difficult, and least understood, challenges a rehabilitation team must face. Researchers and clinicians have explored interventions that can foster awareness in clinical and ecological settings, thus promoting engagement with rehabilitation (Fleming and Ownsworth, 2006). Awareness problems are a key signature of patients with ACoA strokes. This is clearly exemplified by Mrs. B. She understood that something happened to her, intellectually speaking, but she did not feel the stroke generated any changes. In other words, there was intellectual knowledge, but no link between this knowledge and her difficulties in everyday life, the online awareness of cognitive problems when occurring or the anticipation of difficulties. Energization problems added a complex variable to this presentation, since patients with energization impairments experience a decrease in emotional reactivity, compromising the possibility of generating the necessary “emotional noise” or “emotional discrepancy” that often mobilizes behavior (Salas et al., 2014; Balchin et al., 2017). Even though there are several lines of intervention for people with awareness issues (for a review see Fleming and Ownsworth, 2006), the case of Mrs. B is a clear example of how personally meaningful projects can foster awareness. As noted by several authors (Salas, 2009; Feeney and Capo, 2010), engaging patients in meaningful projects increases their permeability to become aware of cognitive and behavioral difficulties. Because they care about the goal they are after, impairments become an obstacle they are willing to contemplate and address, they become emotionally more *salient*. Mrs. B did not engage in, or identify with, tasks related to household management. This, for she did not recognize herself as a housewife. However, when attempting to perform tasks related to her professional identity, Mrs. B the engineer, began noticing her difficulties and considering the explanations proposed by the therapist.

### Using Psychological Interventions to Promote Awareness and Acceptance

Psychological interventions are a core component of contemporary models of Neuropsychological Rehabilitation (Wilson et al., 2009; Yeates and Ashworth, 2019). Several authors have argued that psychological interventions should be considered in the rehabilitation of people with neurobehavioral disorders (Prigatano and Salas, 2017) and energization/initiation problems (Ryan and Yeates, 2021). At an individual level, psychological interventions can promote the understanding of the many changes generated by the injury as well as its impact on personal identity. The case of Mrs. B is particularly enlightening here, since a major consequence of her injury, and impaired functionality, was the loss of individual and relational aspects of her identity. Individually speaking she felt she was no longer the independent and professional woman, perceiving herself now as someone “lazy” and “not in control of her life.” Her family–particularly her children- tended to interpret these changes as laziness and childishness, often becoming critical of Mrs. B and forcing her to follow a routine. In response to this change in roles and power inside the family, Mrs. B often rebelled, and conflict escalated.

An important aspect of the psychological work with Mrs. B’s family was to explore the multiple and diverse experiences of its members (Klonoff, 2014; Clark-Wilson and Holloway, 2019). This aspect of the rehabilitation required to offer education regarding invisible and behavioral impairments as well as a space to think about how they have changed as a family, and how they could sustain intimate relationships with this “new” wife and mother (Bowen et al., 2010; Yeates and Daisley, 2018; Yeates and Salas, 2021). Regarding education, memory problems were difficult to understand and accept by the family, particularly due to its fluctuant presentation. It required time and the occurrence of specific events to generate changes in this front. One day, for example, Mrs. B’s husband arrived at the session surprised that his wife was not able to recall that her new granddaughter had been born! Regarding relational change, it is interesting to note that some of Mrs. B’s children were more tolerant with her impairments and emphasized positive aspects of this new mother. Her daughter, for example, valued that now Mrs. B had time to spend with her grandchildren. Others described that Mrs. B was less stressed and able to enjoy simple things in life. Following the guidelines proposed by Bowen et al. (2010) family work focused on sharing the diverse experiences that each member of the family had, and discussing how they reflected variations in the grief process of each member. This aspect of the rehabilitation process was extremely relevant to contribute to the positive reconstruction of Mrs. B’s identity.

## Conclusion

Patients with ACoA strokes pose an important challenge to rehabilitation teams due to a particular mix of cognitive and behavioral impairments. These deficits compromise engagement with rehabilitation, learning and generalization. In this article we have attempted to describe the many challenges faced by a rehabilitation team when working with one of these patients. Based on those challenges, we propose that the neuropsychological rehabilitation of these patients demands from rehabilitation teams to consider five key elements: (a) to acknowledge the slow pattern of recovery due to anosognosia and memory/executive problems, thus, designing long term interventions; (b) consider the interaction between cognitive and behavioral impairments; (c) keep in mind the relevance of physical and social environments, and their modification to promote functionality and independence; (d) use personal projects to tackle awareness problems and train compensatory skills; (e) employ psychological and relational interventions to promote education and positive identity reconstruction.

This model is a necessary first step in the development of a framework that can help rehabilitation professionals in the design of individual and family interventions. These five principles are intended to work as pillars that sustain clinical reasoning and goal setting, allowing enough flexibility to adapt interventions to the particular profile and needs of each patient, but also providing enough structure to guide interventions in the right direction and wisely distribute economic and emotional resources. This last point is extremely important and deserves to be discussed further. Working with people that have suffered an ACoA stroke can be particularly frustrating for the rehabilitation team. The slow pace of recovery, the pervasiveness of impairments and the altered engagement with the rehabilitation process due to anosognosia, can generate intense negative feelings in the staff, progressively denting the working alliance. It is well known that negative emotional reactions in the rehabilitation team are common when working with brain injured survivors, particularly with people that exhibit neurobehavioral disorders (McLaughlin and Carey, 1993; Judd and Wilson, 2005). In this scenario, a therapeutic model, or a set of guiding principles, can buffer such negative impact, allowing professionals to preserve and promote both hope and realism. As noted by Pamela Klonoff in one of her popular mantras, “everything is about the–emotional health–of the team” (Klonoff, 2015). This is particularly true when working with ACoA survivors.

Developing a model to work with people and relatives that have suffered an ACoA stroke has been a necessary task for our team. However, we believe that other rehabilitation teams, in different geographical and economic contexts, may face similar challenges. We hope this article motivates clinicians and researchers to visibilize this subgroup of patients through new case and experimental studies. It has been a surprise to us the lack of attention this group of patients have received, considering the particular signature of their impairments and the complex functional and psychological needs they present. Studies that explore the level of community integration of these patients as well as mental health problems will be extremely welcomed. Similarly, studies that explore the adaptation, and effectiveness, of compensatory strategies and environmental modifications using single case experimental study designs will be highly informative.

## Data Availability Statement

The original contributions presented in the study are included in the article/Supplementary Material, further inquiries can be directed to the corresponding author.

## Author Contributions

All authors contributed in the design, review of the literature, and writing of the manuscript.

## Conflict of Interest

SP-C was employed by Brain and Behavioral Associates, PC. The remaining authors declare that the research was conducted in the absence of any commercial or financial relationships that could be construed as a potential conflict of interest.

## Publisher’s Note

All claims expressed in this article are solely those of the authors and do not necessarily represent those of their affiliated organizations, or those of the publisher, the editors and the reviewers. Any product that may be evaluated in this article, or claim that may be made by its manufacturer, is not guaranteed or endorsed by the publisher.

## Supplementary Material

Supplementary Appendix 1 Memory diary. Mrs. B used a diary to support both episodic and prospective memory.

Supplementary Appendix 2 Project review log. The log was used to register and manage information during complex tasks like reviewing a map. It worked as a working memory space that fixed information.
