# Supplementary figures and images for: A Neuropsychological Rehabilitation Framework to Address Cognitive and Neurobehavioral Impairments After Strokes to the Anterior Communicating Artery
Source: Front Hum Neurosci. 2022 Jun 10;16:808011. doi: 10.3389/fnhum.2022.808011 (PMC9226309; doi:10.3389/fnhum.2022.808011)

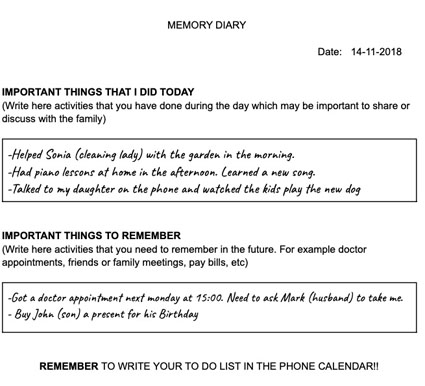

Supplement: Supplementary Appendix 1 — Memory diary. Mrs. B used a diary to support both episodic and prospective memory. [file Image_1.JPEG]

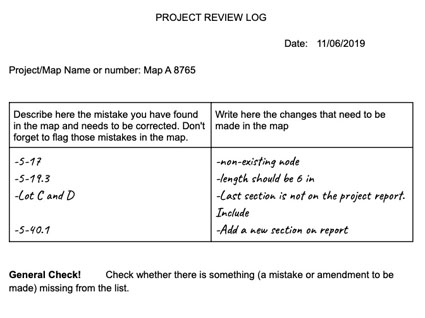

Supplement: Supplementary Appendix 2 — Project review log. The log was used to register and manage information during complex tasks like reviewing a map. It worked as a working memory space that fixed information. [file Image_2.JPEG]
